# Involvement of GLWamide neuropeptides in polyp contraction of the adult stony coral *Euphyllia ancora*

**DOI:** 10.1038/s41598-020-66438-3

**Published:** 2020-06-10

**Authors:** Shinya Shikina, Yi-Ling Chiu, Yan Zhang, Yi-ChenYao, Tai-Yu Liu, Pin-Hsuan Tsai, Céline Zatylny-Gaudin, Ching-Fong Chang

**Affiliations:** 1 Institute of Marine Environment and Ecology, National Taiwan Ocean University, Keelung, 20224 Taiwan; 2 Center of Excellence for the Oceans, National Taiwan Ocean University, Keelung, 20224 Taiwan; 3 Doctoral degree Program in Marine Biotechnology, National Taiwan Ocean University, Keelung, Taiwan; 4 Doctoral degree Program in Marine Biotechnology, Academia Sinica, Taipei, Taiwan; 5 Department of Aquaculture, National Taiwan Ocean University, Keelung, Taiwan; 6 University of Caen-Normandy, 14032 Caen, France

**Keywords:** Neuroscience, Physiology

## Abstract

The existence and function of neurons remain largely unexplored in scleractinian corals. To gain a better understanding of neuronal functions in coral physiology, this study focused on Glycine-Leucine-Tryptophan-amide family neuropeptides (GLWamides), which have been shown to induce muscle contraction and larval metamorphosis in other cnidarians. Molecular identification and functional characterization of GLWamides in the adult stony coral *Euphyllia ancora* were performed. We successfully elucidated the full-length cDNA of GLWamide preprohormone in *E. ancora* (named EaGLW preprohormone). The deduced amino acid sequence was predicted to contain six potential GLWamide peptides. Tissue distribution analysis demonstrated that transcripts of *EaGLW preprohormone* were mainly expressed in the mouth (including the pharynx) and tentacles of the polyps. Immunodetection with an anti-GLWamide monoclonal antibody revealed that GLWamide neurons were mainly distributed in the epidermis of the mouth region and tentacle, in agreement with the distribution patterns of the transcripts. Treatment of the isolated mouth and tentacles with synthetic GLWamide peptides induced the contraction of these isolated tissues. Treatment of polyps with synthetic GLWamide peptides induced the contraction of polyps. These results suggest that GLWamides are involved in polyp contraction (myoactivity) in adult scleractinians. Our data provide new information on the physiological function of neuropeptides in scleractinians.

## Introduction

Neuropeptides are 3- to 70-amino-acid-long linear polypeptides produced by neurons^1,2^. Neuropeptides act either directly or indirectly as modulators and/or hormones and play important roles in various physiological processes (e.g., growth, reproduction, and metabolism) and behavior in animals^1,3–5^. In cnidarians (hydras, sea anemones, jellies, and corals), more than 40 types of neuropeptides have been identified to date, and the significance of neuropeptides in physiological functions at different stages of life has been demonstrated^2,6,7^.

One of the important neuropeptides identified in cnidarians is the glycine-leucine-tryptophan-amide family neuropeptides (GLWamides, also referred to as LWamides)^8^. GLWamides were first isolated from the sea anemone *Anthopleura elegantissima* as a trigger of metamorphosis of *Hydractinia echinata* larvae^9^. Seven types of GLWamides were later isolated from *Hydra magnipapillata*^10^. Some of these GLWamides have been demonstrated to be involved in a wide range of physiological functions, such as larval metamorphosis^11^, muscle contraction (myoactivity)^12^, polyp detachment^10^, and oocyte maturation and ovulation^2,13^, in cnidarians.

GLWamides are derived from a larger precursor molecule called preprohormone (also referred to as prepropeptide). Preprohormone is posttranslationally processed by a series of proteolytic cleavages and modified to yield mature peptides^8,10,14,15^. The mature GLWamides identified in cnidarians to date generally range from 5 to 14 amino acids in length and likely possess conserved structural features in their N- and C-terminal regions^2,8^. For instance, the 7 identified GLWamides in *Hydra* possess a proline residue at the second position (X-Pro) or at the second and third positions (X-Pro-Pro) in their N-terminal region^8,10^. The proline in the N-terminal structure is known to confer resistance to aminopeptidase digestion^16^. In the C-terminal region, GLWamides generally possess glycine-leucine-tryptophan (a GLW motif), and the tryptophan residue in the C-terminus is amidated (-Gly-Leu-Trp-NH_2_).

Scleractinians, also known as stony corals, are ecologically and economically important marine organisms^17^. However, only a few studies on GLWamides have been reported to date. Treatment of planula larvae with a GLWamide peptide (Hym-248, EPLPIGLWamide, identified in *Hydra*) has been shown to induce metamorphosis in specific coral families, such as Acroporidae^18,19^. Although the detailed action mechanisms underlying the induction of metamorphosis have not yet been clarified, GLWamides have been suggested to be involved in the developmental control of embryos in corals^18–20^. Recently, the sequence of GLWamide preprohormone was reported for the first time in *Acropora millepora*, and its transcript expression pattern during embryonic development was clearly elucidated by *in situ* hybridization^21^. In adult *A. millepora*, although the transcript expression of GLWamide preprohormone was detected by transcriptome analysis^22^, the spatial distribution of GLWamide neurons and its physiological function remain unknown.

The present study attempts to investigate the existence and role of GLWamide neurons in adult corals using the gonochoric stony coral *Euphyllia ancora* as the main experimental animal. This animal has large polyps, which allows us not only to easily observe the changes in the behavioral characteristics of the polyp but also to isolate different parts of polyp tissues. These attributes enable us to investigate the spatial distribution pattern of target transcripts and proteins within the polyp^23^, which also enables us to perform a bioassay to investigate the effects of the target molecules on specific coral tissues. With these advantages, the present study investigated the possible physiological role of GLWamide by examining the spatial distribution patterns of transcripts of GLWamide preprohormone and GLWamide neurons in adult *E. ancora* polyps, and the effects of synthetic GLWamides on isolated tissues and whole polyps were also studied. Additionally, the distribution of GLWamide neurons was also investigated in 5 other scleractinian species.

## Materials and Methods

### Experimental animals

Specimens of *E. ancora* were collected at Nanwan Bay in southern Taiwan (21°57′N, 120°46′E) by scuba diving. The collected corals were maintained until use in a 90 L aquarium at National Taiwan Ocean University (NTOU) on a light cycle of approximately 12.5hL: 11.5hD at 26–28 °C. The collection of *E. ancora* was permitted by the administration office of Kenting National Park (Issue number: 1010006545). Samplings of *Stylophora pistillata*, *Pocillopora damicornis*, *Acropora hyacinthus*, and *Favites pentagona* were conducted by snorkeling at Pitouchiao Park (25°07′N, 120°54′E) near northern Taiwan. These animals were selected because they were abundant at the sampling site. The collection of corals was approved by the Fisheries and Fishing Port Affairs Management Office of the New Taipei city government (issue number: 1063334179). *Cycloseris* sp. were purchased from an aquarium shop in Taipei and were propagated at the New Taipei City Marine Resources Recovery Park. Experiments were performed in accordance with the principles and procedures approved by the Institutional Animal Care and Use Committee, NTOU.

### Identification of a partial sequence of GLWamide preprohormone in E. ancora

The sequence of LWamide preprohormone of the stony coral *Acropora digitifera* (XP_015772272) was retrieved from Genbank, and used as a query sequence for a blast search (BLASTP, cut-off e-value of <1 *e*^−5^) against a transcriptome database of adult *E. ancora* (Shikina and Chang, unpublished data) with CLC Main Workbench (CLC Bio, Aarhus, Denmark). The identified partial sequence containing GLWamide motifs was used for downstream experiments.

### RNA extraction, cDNA synthesis, and the full-length cDNA cloning

The isolated tissues were homogenized with a homogenizer (IKA Ultra-turrax, Sigma-Aldrich, St. Louis, USA) in TRIzol reagent (Invitrogen, Carlsbad, USA) on ice. Total RNA was extracted by following the manufacturer’s protocol. First-strand cDNA was synthesized from 2 μg of DNase-treated RNA using Super Script III reverse transcriptase (Invitrogen). Full-length cDNA was obtained using a rapid amplification of cDNA ends (RACE) kit (SMART RACE cDNA; BD Biosciences Clontech, Franklin Lakes, USA) by following the manufacturer’s protocol and using specific primers for the above-identified sequence (Table 1). The nested PCR products were ligated into the pGEM-T Easy vector (Promega, Madison, USA), transformed into competent cells (*Escherichia coli*, JM109 strain, Promega), and sequenced using an ABI Prism 310 Genetic Analyzer (Applied Biosystems, California, USA).

**Table 1:** List of primers.

Primer ID	PCR type	5’−3’ Sequence	T_m_ (°C)	Amplicon size (bp)
EaGLWamide ORF F	RT-PCR	AGGGGAATGTCGCTACGT	45	1149
EaGLWamide ORF R	RT-PCR	GCGAATGTTTATTTGCTT	45	—
EaGLWamide 5′	5′ RACE	GTACTGCTCGCCCCCAGAGTCCTGGTGG	68	643
EaGLWamide 5′ nested	5′ RACE	CATCTTCCGTTTCTTCGTCCTCTTCGCC	62	568
EaGLWamide 3′	3′ RACE	CCACAGAAGGTTGACGAATCGGACGAAG	62	769
EaGLWamide 3′ nested	3′ RACE	GGCGAAGAGGACGAAGAAACGGAAGATG	62	691
EaGLWamide F1	Quantitative RT-PCR	GTTGACGAATCGGACGAAGA	60	157
EaGLWamide R1	Quantitative RT-PCR	AGAGTCCTGGTGGTGCTCTGAT	60	—
β-actin F	Quantitative RT-PCR	CGCCTTCCTTGGAATGGAATCCTCT	60	151
β-actin R	Quantitative RT-PCR	CTGCATCCTGTCAGCGATTCCAGG	60	—

aF and R primers were paired for reverse-transcription PCR (RT-PCR). Rapid amplification of cDNA ends (RACE) primers were nested, with primer 1 used before primer 2 in the sequential amplification reactions.

### Sequence analysis and alignment

The presence of a signal peptide was predicted using PrediSi (http://www.predisi.de/). The EaGLWamide preprohormone was analyzed using Peptraq software to predict the mature peptides^24^. A subset of sequences of LWamide family members from different cnidarians were retrieved from GenBank. The sequence identities were measured by pairwise comparison using Clustal W. Multiple sequence alignment was performed using BioEdit software (http://www.mbio.ncsu.edu/BioEdit/bioedit.html).

### Quantitative reverse transcription (qRT)-PCR analysis

Four different polyp tissues (the tentacle, mouth with pharynx, gonads, and mesenterial filament) were isolated under a stereomicroscope (SZX16; Olympus, Tokyo, Japan) and kept at −80 °C before use. RNA extraction and cDNA synthesis were conducted as described above. The transcript levels of *EaGLWamide preprohormone* in each sample were examined by qRT-PCR analysis. The qRT-PCR amplification was conducted using Bio-Rad thermal cycler (Bio-Rad Laboratories, Hercules, USA) with qPCR SYBR Green Master Mix (Bio-Rad Laboratories) according to the manufacturer’s instructions. Amplification was performed under the following conditions: one cycle of 95 °C for 5 min, followed by 40 cycles of 95 °C for 15 s and 60 °C for 1 min. *E. ancora β-actin* (GenBank accession No. JQ968408) was used as a reference gene. Specific primer pairs (Table 1) were designed using Primer Express 3.0 software (Applied Biosystems). The primer efficiency was determined by 5-fold dilution series of the template cDNA, and only primer pairs that worked at 90–100% efficiency were used for the analysis. Bio-Rad iQ5 Manager (Bio-Rad Laboratories) was used for data analysis according to the 2−ΔΔCt method^25^.

### Whole-mount immunodetection

The tentacles and parts of the mouth, including the pharynx, were isolated and fixed with 20% Zinc Formal-Fixx (Thermo Shandon, Pittsburgh, USA) at room temperature for 16 h. The fixed samples were dehydrated with a series of ethanol solutions for 20 min each (70%, 80%, 90%, 100% twice) at room temperature and stored in 100% methanol at −20 °C before use. The specimens were treated with 50% xylene/50% methanol for 20 min and then 100% xylene for 16 hours at room temperature. After rehydration with a series of ethanol solutions (100%, 80%, 50%, 30%, 0%, for 20 min each), the samples were incubated for 30 min with HistoVT ONE (Nacalai Tesque, Inc., Kyoto, Japan) for antigen retrieval. Blocking was performed using 2% bovine serum albumin (BSA) with phosphate buffered saline (PBS) containing 0.1% Tween 20 (PBT) for 16 hours. A monoclonal antibody against GLWamide (kindly provided by Dr. Toshio Takahashi from the Suntory Foundation for Life Sciences Bioorganic Research Institute) was diluted (1:500) with PBT containing 0.5% BSA and 5% DMSO and was used for the primary reaction. After washing with PBT and blocking with 2% BSA/PBS for 16 hours, the secondary antibody reaction was performed with an alkaline phosphatase-conjugated goat anti-mouse IgG antibody (AnaSpec, San Jose, USA) diluted (1:4,000) in PBT containing 2% BSA. After washing with PBT, visualization of immunoreactivity was performed using the NBT/BCIP liquid substrate system (Sigma-Aldrich, St. Louis, USA).

### Histological and immunohistochemical analysis

Histological and immunohistochemical analyses were carried out according to previously described methodologies^26^. A monoclonal antibody against GLWamide was diluted in PBT (1:500) with 2% skim milk and used for the primary antibody reaction. A biotinylated goat anti-mouse IgG antibody (Vector Laboratories, Burlingame, USA) was diluted 1:4,000 with PBT and 2% BSA and used for the secondary antibody reaction. The sections were then incubated in avidin–biotin–peroxidase complex (ABC) solution (Vector Laboratories), and the immunoreactive signals were visualized by 3,3′-diaminobenzidine (DAB; Sigma-Aldrich). To verify the specificity of the antibody, the anti-GLWamide antibody was preadsorbed with 1 μg/ml of synthetic PPGLWamide (PPGLW-NH_2_) or PPGLW-OH peptide and used for the primary antibody reaction. All other procedures were performed in a manner similar to the immunohistochemical protocols.

### Effects of GLWamide treatment on isolated *E. ancora* tissues

All the peptides used in this study (purities >90%) were synthesized by a local company in Taiwan (Yao-Hong Biotechnology, Inc., Taipei, Taiwan). The tentacles, pieces of mouth with pharynx, and mesenterial filaments were isolated from the dissected *E. ancora* polyp under a stereomicroscope (SZX16; Olympus). The isolated tissues were rinsed three times with 0.22 μm filtered seawater (FSW) to remove excess mucus and were then treated with control vehicle (H_2_O) and 0.1, 1, 10, and 100 μM synthetic PPGLWamide (CONH_2_) peptide in a 48-well plate. To investigate the specificity of the effect of C-terminal extremity of PPGLWamide, the PPGLW-OH (COOH) and PPGLLamide (CONH_2_) peptides were also synthesized and used in treatment experiments for comparison. The treatment experiment was performed 5 times using 5 different colonies. During the treatment, the samples were photographed to monitor distinct changes in morphology or behavior (e.g., contraction) under the stereomicroscope, and the percentage of tissues exhibiting distinct contraction (or crumpling) was determined. Each trial was conducted by two persons to minimize observer bias. The presence or absence of contraction was determined according to the observation results of these two observers. The treatment was determined as effective only if the observation result was consistent between the two observers. Six to ten tissues were isolated from each colony and were used for each trial.

### Effects of GLWamide peptides on *E. ancora* polyps

The fragments of each *E. ancora* colony were placed in buckets with 2 liters of FSW and were maintained for 3–4 hours until their tentacle expanded. To investigate the effects of GLWamide peptides, corals were treated with PPGLWamide, PPGLW-OH, or PPGLLamide by adding these peptides to the culture seawater at a final concentration of 10 μM. Equal volumes of vehicle (H_2_O) were also added to the control group. For the identification of the functional sequence within the PPGLWamide peptide, the effects of 10 μM PPGLWamide, PGLWamide, GLWamide, LWamide, or Wamide peptides on polyp contraction were also investigated. The status of polyp contraction was recorded by taking photographs before treatment and 30 sec, 10, 20, 40, and 60 min after treatment with a digital camera (RX100II, Sony, Tokyo, Japan) that was placed on a height fixing device. Quantification of polyp contraction was conducted by comparing the area of tentacle (polyp) coverage before and after treatment using Image J64 software (National Institutes of Health, Bethesda, MD). Polyp contraction (%) was quantified using the formula (%) = 100 × [Tx/T0], where T0 is the area of tentacle coverage before treatment and Tx is the area of tentacle coverage after treatment at each time point. The experiments were performed three times using 3 different colonies.

### Statistics

The data are shown as the mean ± standard error (SE). Statistical Package for the Social Sciences (SPSS) software was used for analysis of statistical significance with one-way analysis of variance (ANOVA), followed by Tukey’s or Duncan’s test. The statistical significance level was set to P < 0.05.

## Results

### Elucidation of the full-length cDNA encoding EaGLWamide preprohormone, sequence analysis, and sequence comparison

**Figure 1:**
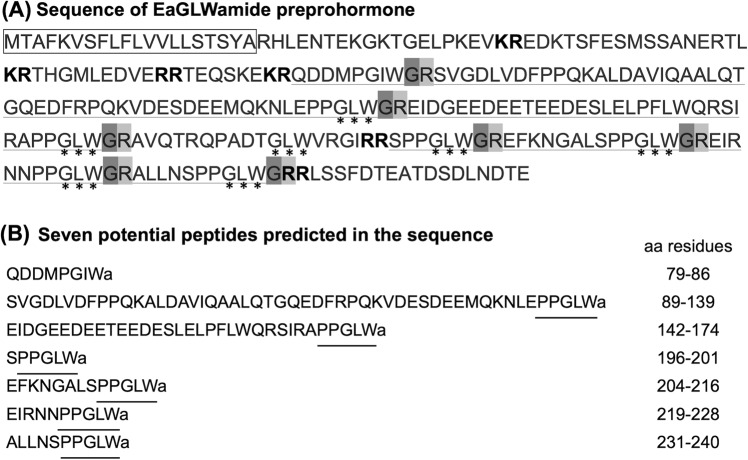
(**A**) Deduced amino acid sequence of EaGLWamide preprohormone. *black box*, predicted signal peptide. *KR/RR*, predicted dibasic cleavage sites; *GR*, C-terminal amidation with a monobasic cleavage site; *underline*, location of potential GLWamide peptide sequences; *asterisk*, GLW motif. (**B**) The seven potential peptides predicted from the EaGLWamide preprohormone using Peptraq software. The positions of the potential peptides in EaGLWamide preprohormone are shown on the right (aa residue). Note that all the potential GLWamide peptides contain a tandem proline residue followed by the GLW motif at the C-terminal end (underlined).

The full-length cDNA for EaGLWamide preprohormone determined by RACE-PCR was 1,231 bp in length and contained an open reading frame of 780 bp corresponding to 260 amino acids (Fig. S1). The deduced amino acid sequences contained a signal peptide at the N-terminal end for protein translocation to the endoplasmic reticulum, 7 GLW motifs, 6 potential dibasic cleavage sites (*KR/RR*), and 7 C-terminal amidations with monobasic cleavage sites (*GRs*) (Fig. 1A). Sequence analysis with Peptraq software predicted six potential GLWamide peptides and single GIWamide peptide (Fig. 1B). All of the sequences of the potential GLWamide peptides contained tandem proline residues followed by GLW motifs at their C-terminal end (Fig. 1B).

**Figure 2:**
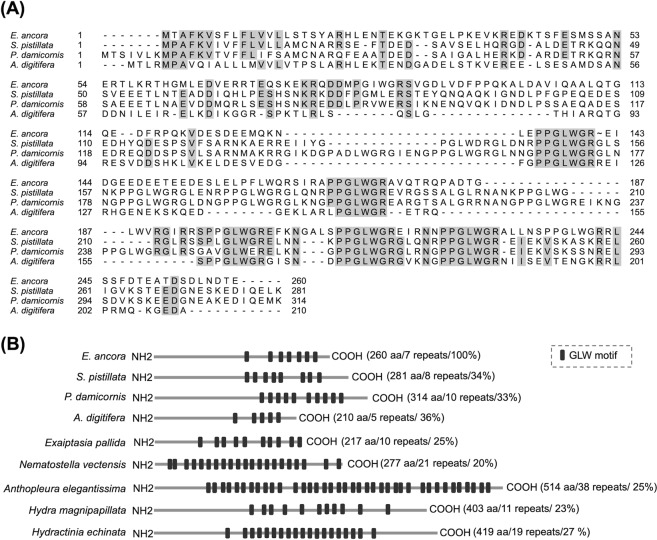
(**A**) Multiple sequence alignments of the deduced amino acid sequences of GLWamide preprohormones from *E. ancora* (this study, QAU55050), the stony corals *Stylophora pistillata* (predicted protein, XP_022779355), *Pocillopora damicornis* (predicted protein, XP_027060554), and *Acropora digitifera* (predicted protein, XP_015772272). Identical residues are indicated in gray. The identity threshold was set to 75%. Gaps introduced into the sequences to maximize homology are indicated by hyphens. (**B**) Comparison of GLWamide preprohormones among different cnidarians. The lengths, repeat numbers of the GLW motif, and sequence identities of the amino acid sequence of EaGLW preprohormone are shown in parentheses. The position of the GLWamide motif is shown as a black box. In addition to the sequences used in the multiple alignment above, sequences from the sea anemones *Exapitasia pallida* (predicted protein, KXJ28092), *Nematostella vectensis* (predicted protein, XM_001626406), *Anthopleura elegantissima* (Q16992), the freshwater Hydra *Hydra magnipapillata* (XP_002164748), and the marine hydroid *Hydractinia echinata* (X89734) were used for the comparison.

Sequence alignment of EaGLWamide preprohormone with GLWamide (also called LWamide) preprohormones from three other scleractinians showed that the sequence identities were low, ranging from 33 to 36%. However, the sequence “PPGLWGR”, which is composed of tandem proline residues followed by a GLWmotif and the amidation-monobasic cleavage signal (GR), was highly conserved among the scleractinians (Fig. 2A,B). Other sequences were varied greatly. In addition, a sequence comparison with other cnidarians, including scleractinians, showed that the sequence length and the numbers of GLW motifs in the sequences largely varied among species (Fig. 2B). The sequence identities between EaGLWamide preprohormone and GLWamide preprohormones of other cnidarians, such as sea anemones, *Hydra*, and *Hydractinia*, were low, ranging from 20 to 27%.

### Tissue distribution of the transcripts of EaGLWamide preprohormone in the polyp

**Figure 3:**
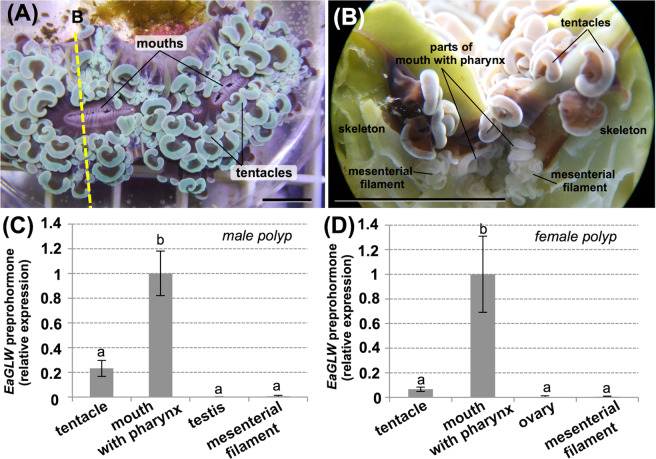
(**A**) Representative picture of *E. ancora* in an aquarium (viewed from above). Mouths are at the middle of the polyps, and tentacles surround the mouths. Bar, 1 cm. (**B**) Representative picture of a polyp, including the skeleton, dissected through the mouth at the position of the yellow-broken line shown in (A). Bar, 1 cm. (**C**) Expression levels of *EaGLW preprohormone* transcripts in different types of male polyp tissues. (**D**) Expression levels of *EaGLW preprohormone* transcripts in different types of female polyp tissues. For this analysis, 4 different parts of polyp tissues (the tentacle, mouth including pharynx, gonad, and mesenterial filament) were microscopically isolated, and the expression levels of the *EaGLW preprohormone* transcripts were investigated by quantitative RT-PCR analysis. Data are shown as the mean ± SE (4 male or 3 female colonies) of the relative values of the mouth group. *E. ancora β-actin* was used as the reference gene. Groups with different letters are significantly different (P < 0.05).

Next, the transcript distribution of *EaGLWamide preprohormone* in *E. ancora* polyps was determined (Fig. 3A,B). The 4 different polyp tissues—the tentacle, mouth including the pharynx, gonads (testis/ovary), and mesenterial filament—were microscopically isolated from polyps of both sexes, and the transcript levels of *EaGLW preprohormone* were compared by qRT-PCR analysis. The transcripts were significantly highly expressed in the mouth, including the pharynx, compared with their expression in other tissues in both male (Fig. 3C) and female (Fig. 3D) polyps. The tentacles also had high but not significant levels of transcripts in both sexes. Control reactions lacking either reverse transcriptase or template cDNA did not yield a product (data not shown).

### Identification of GLWamide neurons in *E. ancora* polyps

**Figure 4:**
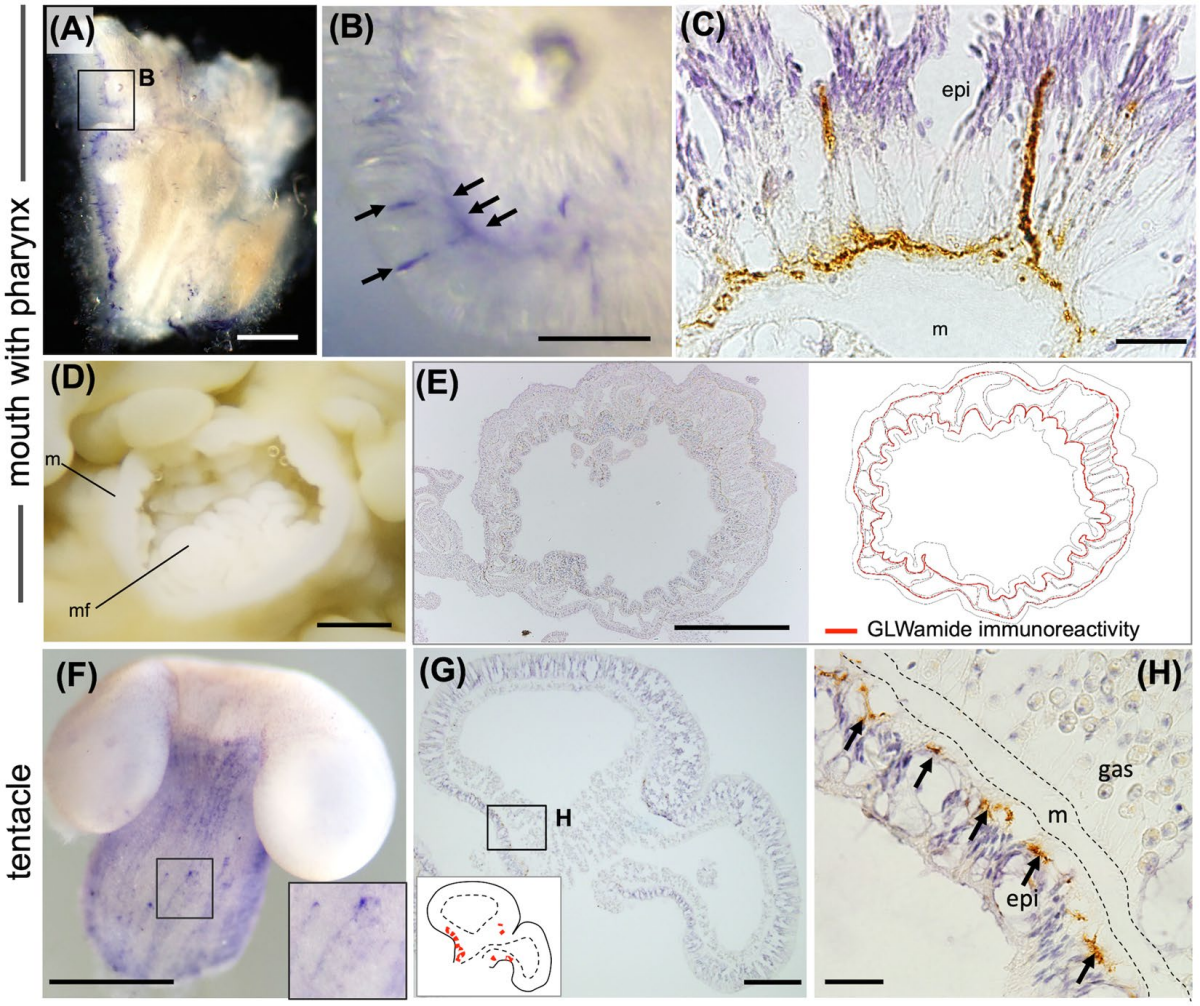
Detection of GLWamide neurons in the mouth (with pharynx) and tentacle of *E. ancora*. (**A**) Whole-mount immunodetection of GLWamide neurons in an isolated piece of mouth including the pharynx. (**B**) Higher-magnification view of the inset in (**A**). Arrows indicate GLWamide neurons exhibiting immunoreactivity (purple color). (**C**) Immunohistochemical detection of GLWamide neurons (brown color) in the mouth. (**D**) Representative picture of the mouth located at the middle of the polyp (above view). Note that the polyp became cream-colored due to fixation and preservation in ethanol. m, mouth; mf, mesenterial filament. (**E**) Immunohistochemical detection of GLWamide neurons in the identical mouth sample shown in (**D**). The schematic illustration on the right shows the distribution of GLWamide neurons (red line). (**F**) Whole-mount immunodetection of GLWamide neurons in an isolated tentacle. The inset shows a higher magnification of the area indicated by the arrows. Cells exhibiting immunoreactivity (purple color) were observed. (**G**) Immunohistochemical detection of GLWamide neurons (brown color) in the tentacle. The inset on the left shows a schematic illustration depicting the distribution of GLWamide neurons (red spots). (**H**) Higher-magnification view of the inset in (**G**). Arrows indicate the GLWamide neurons exhibiting immunoreactivity (brown color). m, mesoglea; epi, epidermis; and gas, gastrodermis. Scale bars = 500 μm (**A**); 100 μm (**B**,**C**); 1 mm (**D**,**E**,**F**); 200 μm (**G**); 20 μm (**H**).

Immunodetection with a monoclonal antibody against GLWamide was performed to identify GLWamide neurons in the coral polyp. In the isolated pieces of the mouth with the pharynx, GLWamide neurons were mainly detected in the epidermis along the mesoglea (Fig. 4A-C). Immunohistochemical analysis showed that GLWamide neurons were distributed in the forms of a nerve ring (Fig. 4D,E, Fig. S2). In the isolated tentacle, GLWamide neurons were detected in the column but not in the apical region, which has a number of cnidoblasts (Fig. 4F). Histological analyses showed that GLWamide neurons were localized in the tentacle epidermis along the mesoglea (Fig. 4G,H). Only a few immunoreactive cells were detected in other tissues, such as the testis, ovary, and mesenterial filaments (Fig. S3). The immunoreactivity of GLWamide in the mouth and tentacle was abolished by preabsorption of the anti-GLWamide antibody with GLWamide (PPGLWamide) before the primary antibody reaction (Fig. S4A-H).

### GLWamide neurons in the adult polyps of other scleractinians

GLWamide neurons were also mainly detected in the mouth regions, including the pharynx of other adult polyps of scleractinians, including the Pocilloporidae families (*S. pistillata*, *P. damicornis*), Acroporidae (*A. hyacinthus*), Merulinidae (*F. pentagona*), and Fungiidae (*Cycloseris* sp.) (Table 2 and Fig. S5). The number of observed immunoreactivities in the tentacles varied among species. Only a few immunoreactivities were detected in other tissues, such as mesenterial filaments (Table 2).

**Table 2:** Distribution of GLWamide neurons in scleractinian corals as assessed by immunohistochemical analysis.

Order	Family	Species name	Immunoreactivity			
			Mouth with pharynx	Tentacle	Mesenterial filament	Gonad
Scleractinia	Euphyllidae	*Euphyllia ancora*	+++	++	+	+
	Pocilloporidae	*Stylophora pistillata*	+++	+	+	unknown
	Pocilloporidae	*Pocillopora damicornis*	+++	+	+	unknown
	Acroporidae	*Acropora hyacinthus*	+++	+	+	unknown
	Merulinidae	*Favites pentagona*	+++	++	+	unknown
	Fungiidae	*Cycloseris* sp.	+++	++	+	unknown

The levels of number of cells exhibiting immunoreactivity were defined as four grades: many (+++), moderate (++), a few (+), and negative (-).

### Effects of GLWamides on *E. ancora* isolated tissues and polyps

**Figure 5:**
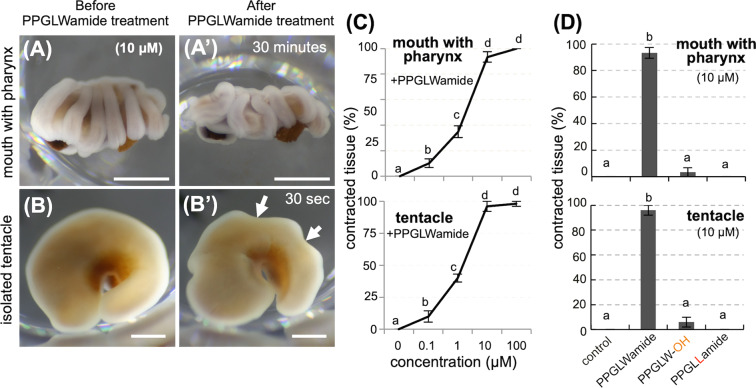
Effects of PPGLWamide peptides on isolated pieces of the mouth (with the pharynx) and tentacles. (**A**) An isolated piece of mouth with the pharynx before treatment. (A’) Identical tissue at 65 minutes after treatment with 10 μM PPGLWamide peptide. (**B**) An isolated tentacle before treatment. (B’) Identical tissue at 1 minute after treatment with 10 μM PPGLWamide peptide. Note that the tissues treated with PPGLWamide peptide exhibited contraction. Arrows indicate dents observed on the tentacle. Scale bars= 1 mm. See also Fig. S6 and S7. (**C**) The effects of PPGLWamide peptide (0-100 μM) on the isolated piece of mouth with the pharynx. (**D**) The effects of treating the isolated piece of the mouth with control (vehicle, H_2_O), 10 μM PPGLWamide, 10 μM PPGLW-OH, or 10 μM PPGLLamide peptide. For (**C**,**D**), the percentages of isolated tissues exhibiting a distinct contraction after treatment (contracted tissues) were determined. Data are shown as the mean ± SE (n = 5 experiments). Groups with different lower case letters are significantly different (P < 0.05).

Because all of the predicted GLWamides in *E. ancora* commonly contained tandem proline residues followed by the GLWmotif (PPGLW) at the C-terminal end (Fig. 1B) and because the sequence was highly conserved among scleractinians (Fig. 2A), we synthesized PPGLWamide peptide and used it in a treatment experiment. As controls, the PPGLW-OH peptide (a nonamidated analogue) and PPGLLamide peptide were also synthesized and used in the experiment. A pilot experiment with 10 μM PPGLWamide peptide showed that the treatment could induce the contraction of an isolated piece of mouth (including the pharynx) and tentacles within 30 min and 30 sec after treatment, respectively (Fig. 5A, A’, B, and B’; Fig. S6 and S7). Treatment with different doses of PPGLWamide peptide showed that 10 to 46% of the isolated piece of mouth (including the pharynx) and tentacles receiving 0.1 and 1 μM PPGLWamide showed contraction (Figs. 5C). At higher doses of PPGLW (10 and 100 μM), 93–100% of the isolated tissues showed apparent contraction (or crumpling) (Fig. 5C). Tissue contractions were not observed under treatment with vehicle H_2_O (control) or 10 μM PPGLLamide in either isolated pieces of the mouth (including the pharynx) or tentacles (Fig. 5D). Three to six percent of the isolated tissues that received 10 μM PPGLW-OH peptide showed contraction (Fig. 5D). GLWamide treatment did not induce contraction or any other apparent changes in the isolated mesenterial filaments (data not shown).

**Figure 6:**
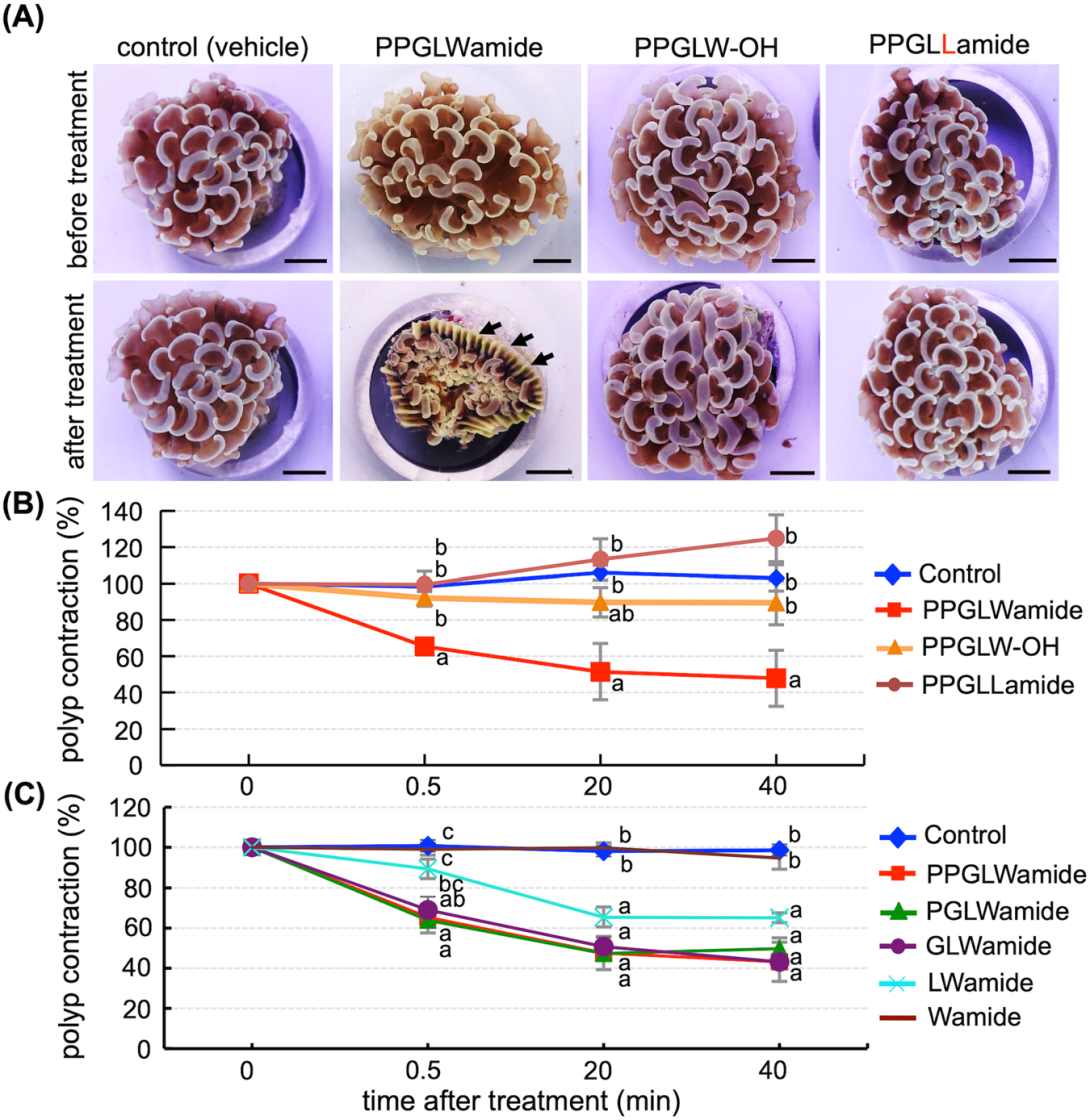
Effects of PPGLWamide peptide on *E. ancora* polyps. (**A**) Representative pictures of *E. ancora* before and after treatment with control (vehicle, H_2_O), 10 μM PPGLWamide peptide, 10 μM PPGLW-OH peptide, or 10 μM PPGLLamide peptide. The top panels show the *E. ancora* polyps before treatment, and the bottom panels show the identical *E. ancora* polyps at 40 minutes after treatment. Note that only the polyps treated with PPGLWamide peptide exhibited distinct polyp contraction. Arrows indicate the exposed skeleton of *E. ancora*. (**B**) Quantified effects of the treatment with control (vehicle, H_2_O), 10 μM PPGLWamide peptide, 10 μM PPGLW-OH peptide, or 10 μM PPGLLamide peptide on polyp contraction. (**C**) Quantified effects of the treatment with control (vehicle, H_2_O), 10 μM PPGLWamide, 10 μM PGLWamide, 10 μM GLWamide, 10 μM LWamide, or 10 μM Wamide peptides on polyp contraction. In (B) and (C), the rates of polyp contraction (%) were determined by comparing the area of tentacle coverage of *E. ancora* before treatment (0 min) and after treatment (0.5, 10, 20, 40, and 60 minutes). Each data point represents the mean ± SE (n = 3 different colonies). Different lower case letters above the symbols depict significant differences (P < 0.05) among the groups at each time point.

Next, we treated *E. ancora* polyps with GLWamide. The polyps treated with 10 μM PPGLWamide showed distinct contraction, whereas the control polyps treated with vehicle H_2_O, 10 μM PPGLW-OH, or 10 μM PPGLLamide did not show apparent changes (Fig. 6A). Quantification of polyp contraction status by determining the area of tentacle (polyp) coverage showed that effects were observed within 30 sec after the treatment and were maintained for at least 60 minutes (Fig. 6B). In contrast, polyp contraction was not detected in the control groups treated with vehicle H_2_O, 10 μM PPGLW-OH, or 10 μM PPGLLamide peptide during the observation period (Fig. 6B). Subsequently, to identify the functional sequence within PPGLWamide peptide, the polyps were treated with 10 μM PGLWamide, GLWamide, LWamide, or Wamide peptide (Fig. 6C). Polyp contraction was induced by the 10 μM PPGLWamide, 10 μM PGLWamide peptides, and 10 μM GLWamide peptide treatments but not by the vehicle H_2_O and 10 μM Wamide peptide treatments. Moderate polyp contraction was observed in the groups treated with 10 μM LWamide peptide (Fig. 6C).

## Discussion

Although neuropeptides have been shown to play important roles in various physiological processes in cnidarians, the existence and function of neuropeptides remain largely unexplored in scleractinians. With the advantages of *E. ancora*, this study successfully revealed a full-length cDNA sequence encoding *GLWamide* preprohormone and investigated the spatial distribution patterns of its transcripts in the polyps of both sexes. In agreement with the transcript distribution of *EaGLWamide preprohormone*, GLWamide neurons were shown to be present in the mouth (including the pharynx) and tentacles. Furthermore, bioassay systems using isolated tissues and intact polyps showed that the PPGLWamide peptide treatments induced tissue and polyp contraction, suggesting that GLWamides are most likely involved in the myoactivity of adult coral. To the best of our knowledge, the present study is the first to document the spatial localization patterns of GLWamide neurons and their possible physiological function in adult corals.

Sequence comparison and alignment of GLWamide preprohormone from scleractinians have not been described in the literature. Our sequence analysis showed that the only region of the sequences that appeared to be highly conserved was PPGLWGR. We also found that all the predicted GLWamide peptides in *E. ancora* contained the PPGLW sequence at the C-terminal end. Most but not all of the predicted GLWamide peptides in the three other scleractinians (*S. pistillata*, *P. damicornis*, and *A. digitifera*) contained the PPGLW sequence (data not shown). The N-terminal region of the predicted GLWamide peptides was highly variable among species. The selective conservation of the PPGLW sequence suggests its functional significance in coral physiology and thus its low tolerance for sequence alterations during evolution.

To reveal how neurons play roles in each tissue of the polyp, it will be important to understand the spatial distribution patterns of neurons within the polyps. Recently, the distribution of a subpopulation of neurons in adult scleractinian corals was shown in *A. millepora* by immunocytochemical detection with an anti-FMRF antibody^21^. Immunoreactive cells were mainly detected around the mouth, including the pharynx, forming a nerve ring. Immunoreactive sensory cells and a large bundle of immunoreactive neurons were also detected in the mesenterial filaments, which are the core tissues of the digestive system in scleractinians^21^. Our study, for the first time, revealed the distribution characteristics of GLWamide neurons in the adult polyps of various scleractinians. Similar to the FMRFamide immunoreactive cells shown in *A. millepora*, GLWamide neurons were mainly distributed in the mouth, including the pharynx, and formed a nerve ring, suggesting that the neurons that form the neural ring in corals are heterogeneous. The major difference in the distribution patterns between GLWamide neurons and FMRFamide immunoreactive neurons was found in the mesenterial filament where only a few GLWamide signals were detected in our study. Further marker-assisted investigation in polyps to identify specific subpopulations of neurons, together with treatment assays, would provide new insights and allow a better understanding of the physiological function of neuropeptides in scleractinians.

PPGLWamide peptide treatment was shown to induce the contraction of the adult polyp of *E. ancora*. In other cnidarians, such as the sea anemones *A. fuscoviridis* and *Hydra*, the induction of muscle contraction (myoactivity) by GLWamides has also been demonstrated^8,12^. Although the detailed neuron activity under GLWamides treatment should be monitored by an electrophysiological approach in future studies, our findings strongly suggest that GLWamides are involved in the muscle contraction of scleractinians. For scleractinians, polyp contraction is one of the primary physiological responses when they are exposed to both abiotic and biotic stressors (e.g., desiccation and attack of predators). Polyps have been observed to expand/contract in response to daily sunlight patterns in some species^27–30^. Generally, the former contraction is quicker than the latter one. Although the phenomenon of polyp expansion/contraction has been described since the 1930s^31^, the intrinsic regulation mechanism has not been reported in any species. It will be of interest to determine the changes in the transcript levels of GLWamide preprohormone when coral exhibit the diel patterns of polyp expansion and contraction or stress-induced contraction. Knowledge of these changes would provide further insight into the physiological function of GLWamides in adult coral.

Polyp contraction was only observed when the *E. ancora* polyps were treated with the synthetic GLWamide and LWamide peptides but not with PPGLW-OH (a nonamidated analogue), PPGLLamide, and Wamide peptides. Similarly, PPGLWamide peptide treatment induced contraction of the isolated mouth (including the pharynx) and tentacles. These results indicate that C-terminal LWamide is required for the biological activity. It was also suggested that specific receptors mediating GLW (or LW) amides exist in the mouth (including the pharynx) and tentacles of *E. ancora*, and that signal transduction for muscle contraction was induced by the treatment. In *Hydra*, the presence of two types of receptors for GLWamides has been experimentally suggested^8^. However, these receptors have not been identified in *Hydra* or, more generally, in any other cnidarians. For the future identification of receptors for GLWamides in corals, affinity-trapping methods (e.g., biotin-tagged GLWamides) with LC-MALDI-MS/MS^32^ could be applied. The mouth with pharynx tissues can be used as the appropriate material.

Mesenterial filaments are lamella-like extensions of mesenteries in the gastrovascular cavity of polyps. Mesenterial filaments are mainly responsible for digestion and absorption of particulate food^33^. Previous anatomical and ultrastructural analyses of the scleractinian coral *Mycetophylla reesi* showed the existence of cnidocytes, secretory cells, collar cells, muscle cells, and neurons in the mesenterial filaments^34^. During the course of treatment experiments using isolated mesenterial filaments from *E. ancora*, we unexpectedly found that they could survive for at least 1 week in Petri dishes in filtrated seawater without apparent tissue degradation. Isolated mesenterial filaments also exhibited slow movement characteristics with contraction and extension in cultures, suggesting that they could maintain tissue integrity and some physiological properties. However, PPGLWamide peptide treatment did not induce contraction or any other apparent changes in the movement characteristics of isolated mesenterial filaments. Since our immunohistochemical analysis showed that only a few GLWamide immunoreactivities were detected in the mesenterial filament, GLWamides may not be involved in the movement characteristics of the mesenterial filament, and other mechanisms may be involved in its regulation.

Almost nothing is known about the presence of neurons in coral gonads. The present study showed low levels of transcripts of *EaGLW preprohormone* and a few, but not many, GLWamide immunoreactivities adjacent to germ cells in both the testis and ovary of *E. ancora*. These findings raise the possibility that GLWamides may be involved in coral reproduction, such as germ cell development. In the jellyfish *Cytaeis uchidae*, the presence of GLWamide-containing neurons has been demonstrated in the ovary, and GLWamide treatment (Hym-53; NPYPGLWamide) has been shown to induce oocyte maturation and ovulation^13^. Further investigation via GLWamide treatment of the isolated gonads at different points of gametogenesis would reveal the role of GLWamides in coral reproduction in future studies.

Several types of GLWamides have been identified from extracts of *Hydra* polyps by HPLC^10^. Preprohormone cDNA encompassing all of the identified *Hydra* GLWamides has also been cloned^15^. Our sequence analysis with the software Peptraq predicted six potential GLWamide peptides and a potential GIWamide peptide in EaGLW preprohormone. Because the previous study showed that cnidarian preprohormone contain unusual or novel processing sites (such as S or N)^15^, it is probable that the lengths of native peptides are shorter than that of the predicted potential peptides. Although several attempts to isolate these native peptides from the crude extract of *E. ancora* whole-polyp tissue have been made using LC-MS/MS analysis, the identification has not been successful, possibly due to the low concentration of these peptides.

Our expression analysis indicates that the mouth including the pharynx is a suitable sample for the identification of these native peptides. However, because the mouth is small and a large amount of a wild *E. ancora* colony is necessary to prepare an adequate amount of samples for analysis, we have been reluctant to collect samples to conserve the wild *E. ancora* population from an ecological viewpoint. For the future identification of native GLWamides, the establishment of artificial propagation methods of *E. ancora* as well as the usage of an ultrasensitive LC-MS system (e.g., NanoLC-MS/MS system,^35^) would be necessary.

## Supplementary information

Supplementary Information.
