# Supplementary material for: Involvement of GLWamide neuropeptides in polyp contraction of the adult stony coral Euphyllia ancora
Source: Sci Rep. 2020 Jun 10;10:9427. doi: 10.1038/s41598-020-66438-3 (PMC7287070; doi:10.1038/s41598-020-66438-3)
Supplement: Supplementary file 1 — Supplementary Information. [file 41598_2020_66438_MOESM1_ESM.pdf]

Title: Involvement of GLWamide neuropeptides in polyp contraction of the adult stony coral *Euphyllia ancora*

Authors: Shinya Shikina[1, 2*], Yi-Ling Chiu[3,4], Yan Zhang[2,5], Yi-ChenYao[5], Tai-Yu Liu[5], Pin-Hsuan Tsai[1], Céline Zatylny-Gaudin[6], and Ching-Fong Chang[2,5*]

Affiliations: [1]Institute of Marine Environment and Ecology, National Taiwan Ocean University, Keelung, 20224, Taiwan; [2]Center of Excellence for the Oceans, National Taiwan Ocean University, Keelung, 20224, Taiwan; [3]Doctoral degree Program in Marine Biotechnology, National Taiwan Ocean University, Keelung, Taiwan, [4]Doctoral degree Program in Marine Biotechnology, Academia Sinica, Taipei, Taiwan, [5]Department of Aquaculture, National Taiwan Ocean University, Keelung, Taiwan, and, [6]University of Caen-Normandy, 14032 Caen, France.

```
   1 GAGCCAAATTTCTTTTAAAACCATAACGTTAAGGTGCCTTTAGGCAAAGGGGAATGTCGCTACGTTTTTATGTAAGTTAG 80

  81 ATCTTGAACATTTGCACAACAGAAAAGAATGACTGCCTTTAAAGTATCCTTCCTGTTTCTGGTGGTTTTGTTGTCTACTT 160
                                    M  T  A  F  K  V  S  F  L  F  L  V  V  L  L  S  T  S
 161 CTTATGCTCGGCATCTGGAAAATACAGAAAAAGGCAAAACTGGTGAACTGCCAAAGGAAGTGAAAAGAGAAGACAAAACA 240
       Y  A  R  H  L  E  N  T  E  K  G  K  T  G  E  L  P  K  E  V  K  R  E  D  K  T
 241 TCTTTTGAATCCATGAGCAGTGCAAATGAACGGACCCTCAAACGGACCCATGGAATGCTAGAGGATGTAGAACGGAGGAC 320
     S  F  E  S  M  S  S  A  N  E  R  T  L  K  R  T  H  G  M  L  E  D  V  E  R  R  T
 321 TGAACAATCCAAAGAGAAAAGACAAGACGACATGCCTGGTATATGGGGGAGGTCAGTAGGGGATCTAGTTGACTTCCCGC 400
      E  Q  S  K  E  K  R  Q  D  D  M  P  G  I  W  G  R  S  V  G  D  L  V  D  F  P  P
 401 CACAAAAAGCCTTGGATGCCGTTATTCAGGCCGCCCTTCAAACAGGACAAGAAGATTTCCGACCACAGAAGGTTGACGAA 480
       Q  K  A  L  D  A  V  I  Q  A  A  L  Q  T  G  Q  E  D  F  R  P  Q  K  V  D  E
 481 TCGGACGAAGAAATGCAGAAGAACCTTGAACCACCTGGTCTCTGGGGGCGAGAAATTGATGGCGAAGAGGACGAAGAAAC 560
     S  D  E  E  M  Q  K  N  L  E  P  P  G  L  W  G  R  E  I  D  G  E  E  D  E  E  T
 561 GGAAGAAGATGAGAGCCTTGAGCTTCCATTTCTTTGGCAGCGATCGATCAGAGCACCACCAGGACTCTGGGGGCGAGCAG 640
      E  E  D  E  S  L  E  L  P  F  L  W  Q  R  S  I  R  A  P  P  G  L  W  G  R  A  V
 641 TACAAACACGCCAACCAGCAGACACCGGTCTCTGGGTGAGAGGAATAAGACGGAGCCCACCGGGACTCTGGGGAAGGGAA 720
       Q  T  R  Q  P  A  D  T  G  L  W  V  R  G  I  R  R  S  P  P  G  L  W  G  R  E
 721 TTTAAAAATGGTGCACTAAGTCCCCCAGGTCTGTGGGGTCGCGAAATTAGGAACAATCCACCAGGACTCTGGGGTCGGGC 800
     F  K  N  G  A  L  S  P  P  G  L  W  G  R  E  I  R  N  N  P  P  G  L  W  G  R  A
 801 CTTACTGAACAGCCCTCCCGGTCTCTGGGGAAGAAGGCTTTCGAGTTTCGACACAGAAGCCACTGATAGCGATCTTAATG 880
      L  L  N  S  P  P  G  L  W  G  R  R  L  S  S  F  D  T  E  A  T  D  S  D  L  N  D
 881 ACACAGAGTGACCCTGTAGGCAAGCTAATGGATGAACCTTAAAACGAACAAAACGAACGTTACGAATTTTGCTTTCGCTT 960
       T  E  *
 961 TTACAGATCGAAGAGTGTCAATCATGTGAAAGTTGAAATAAATAATGCGTGACATTAAAGCAGGGGTATGTAATAAGTTA 1040

1041 ATGATTTGAGTAGTGAATAGCCTCTCCTTATGTCAAAAACTGTAGTTGTTTACTTATTAGCTAGTGCCAGCAGTCATGTA 1120

1121 TGTTCCAGAAGAGGCAATAGCTGTTCCTTAATTGTACAAATTCTTAAGGGTAAACCTCAAGCAAATAAACATTCGCAATC 1200

1201 ATGGGATGTCAGAAAAAAAAAAAAAAAAAAA 1231
```

Figure S1

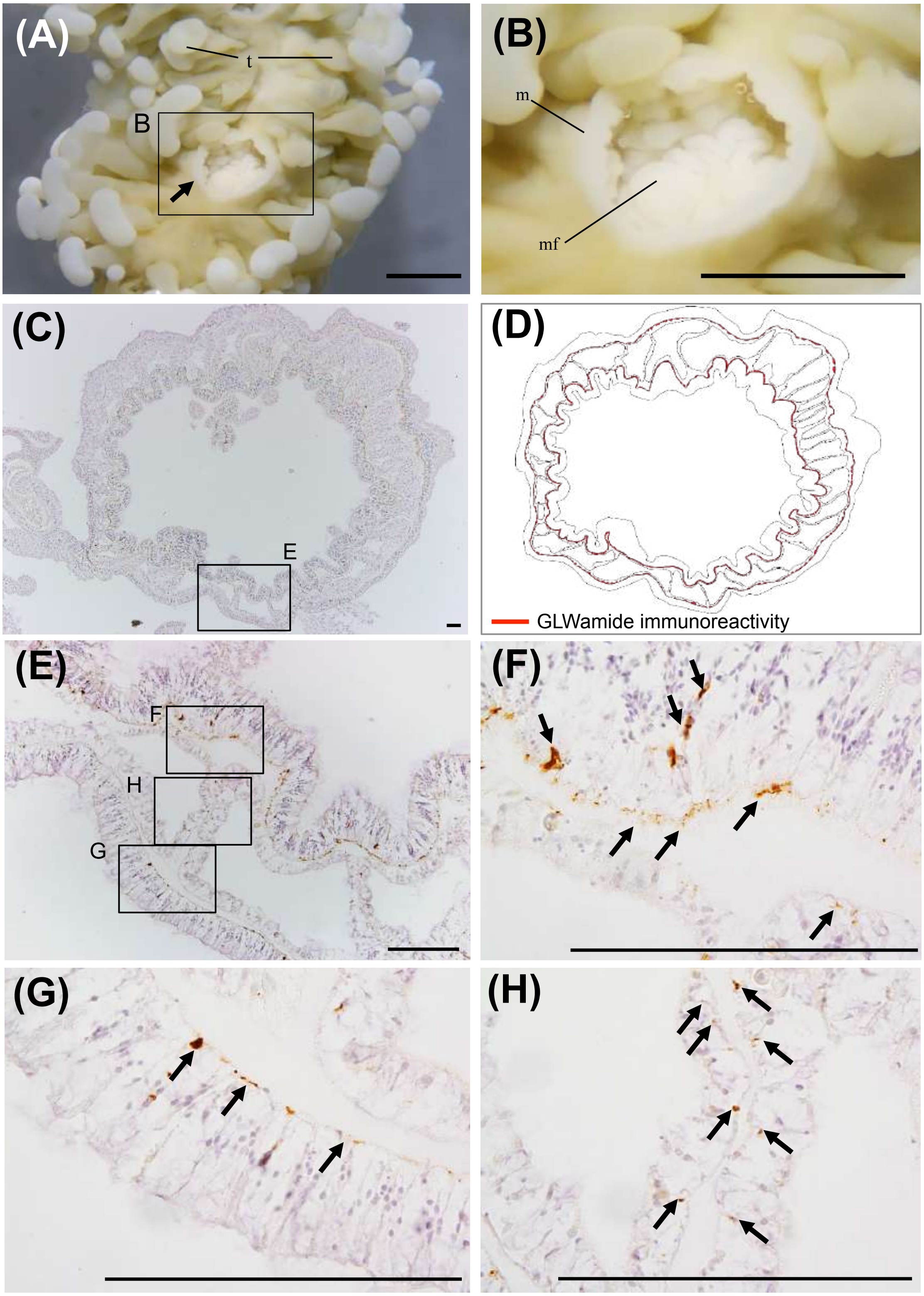

Figure S2

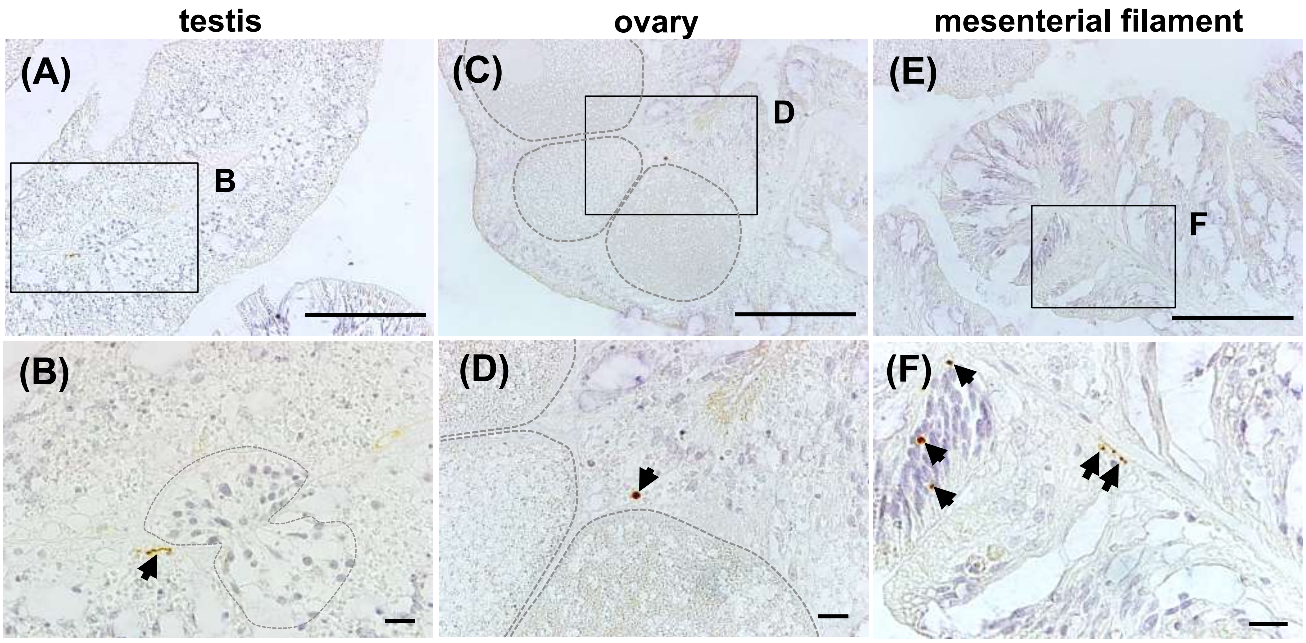

Figure S3

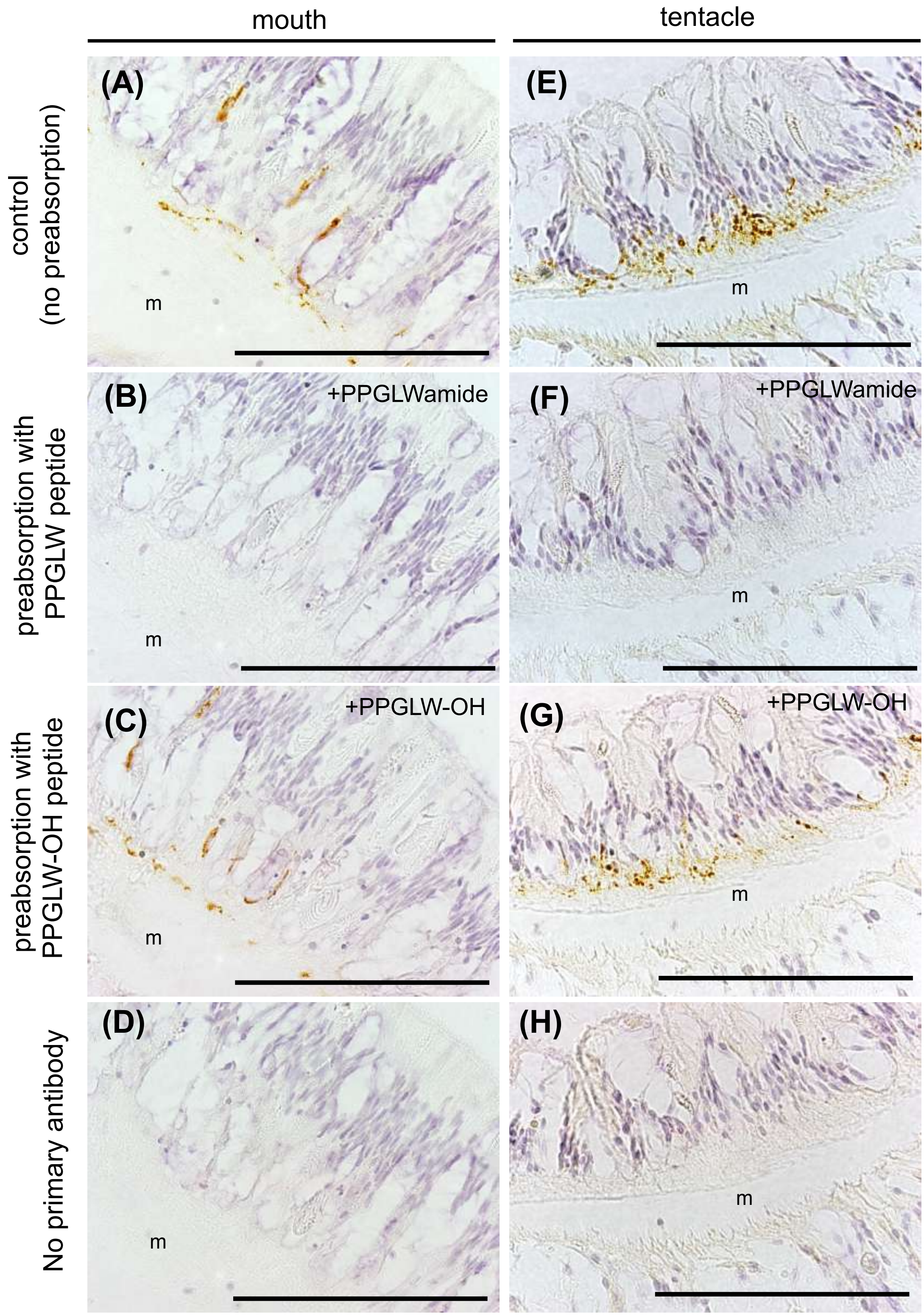
mouth
tentacle
control
(no preabsorption)
preabsorption with
PPGLW peptide
preabsorption with
PPGLW-OH peptide
No primary antibody
(A)
(B)
+PPGLWamide
(C)
+PPGLW-OH
(D)
(E)
(F)
+PPGLWamide
(G)
+PPGLW-OH
(H)
m

Figure S4

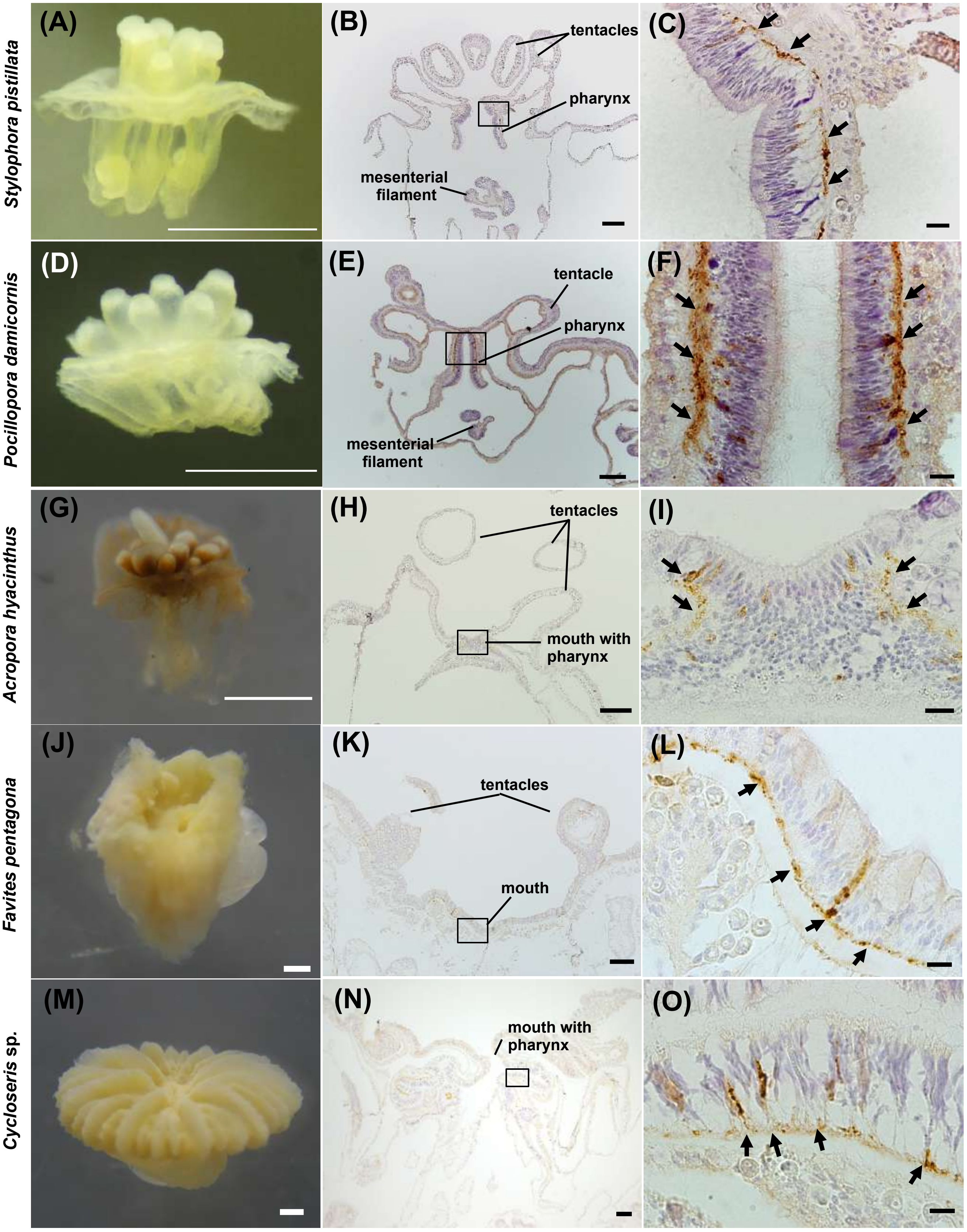
(A)
(B)
tentacles
pharynx
mesenterial
filament
(C)
Stylophora pistillata
(D)
(E)
tentacle
pharynx
mesenterial
filament
(F)
Pocillopora damicornis
(G)
(H)
tentacles
mouth with
pharynx
(I)
Acropora hyacinthus
(J)
(K)
tentacles
mouth
(L)
Favites pentagona
(M)
(N)
mouth with
pharynx
(O)
Cycloseris sp.

Figure S5

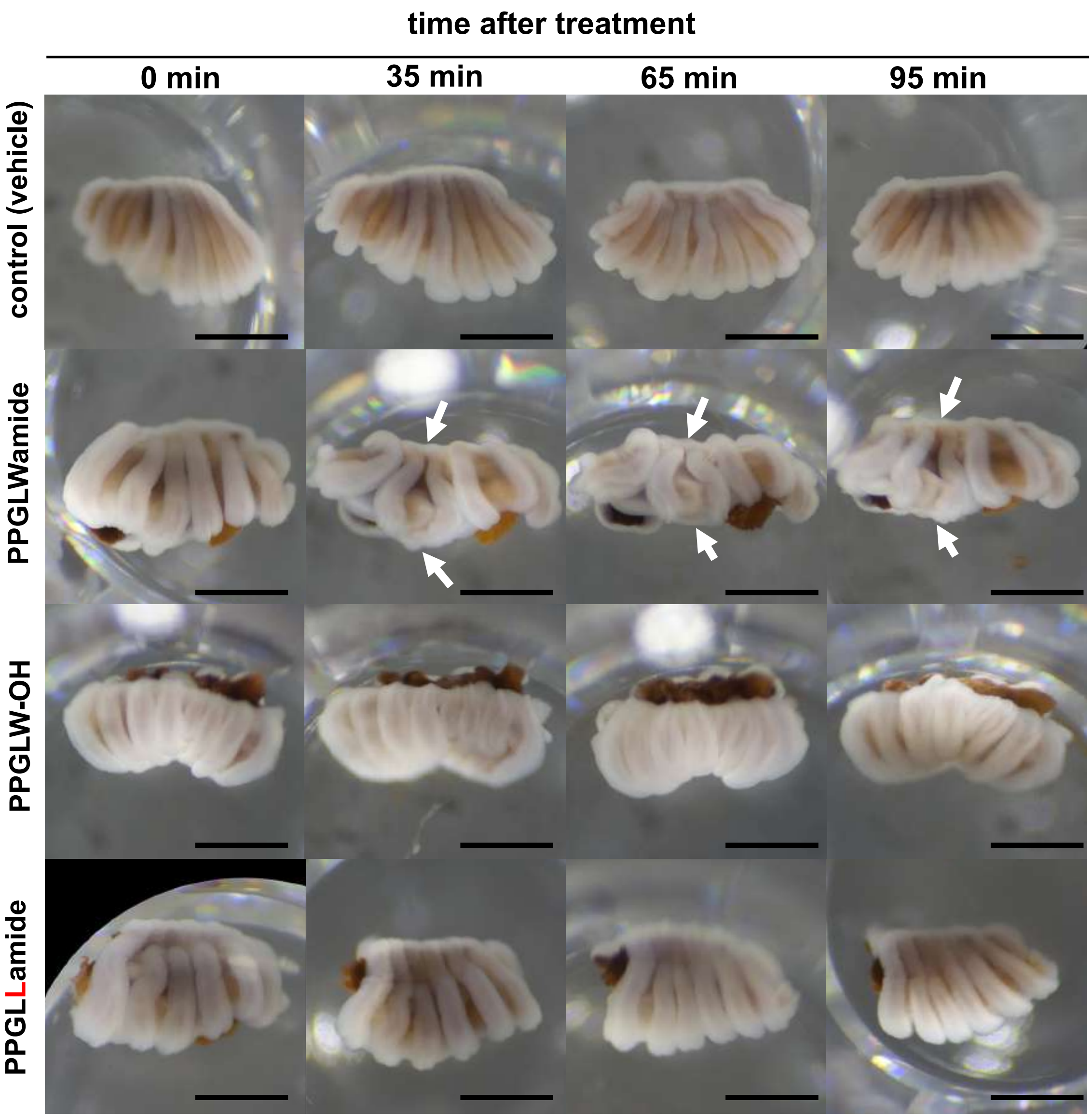
time after treatment
0 min
35 min
65 min
95 min
control (vehicle)
PPGLWamide
PPGLW-OH
PPGLLamide

Figure S6

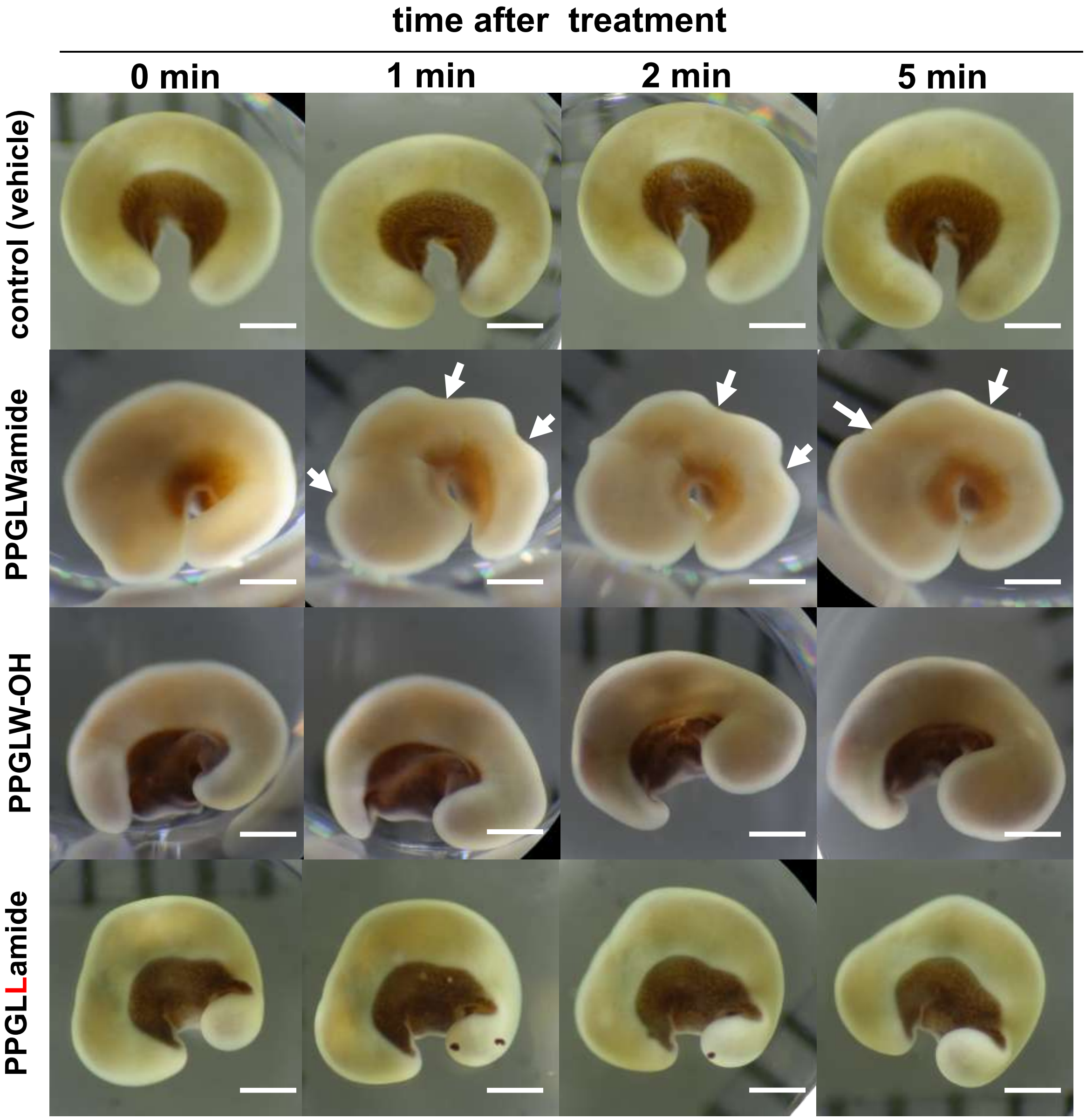
time after treatment
0 min
1 min
2 min
5 min
control (vehicle)
PPGLWamide
PPGLW-OH
PPGLLamide

Figure S7
